# RETRACTION: Effect of Comprehensive Nursing Intervention on Wound Pain and Wound Complications in Patients With Tonsillectomy: A Meta‐Analysis

**DOI:** 10.1111/iwj.70438

**Published:** 2025-03-26

## Abstract

**RETRACTION:** WuJ.‐L. , ZhangQ. , ZhangL.‐H. , and LiJ.‐Y. , “Effect of Comprehensive Nursing Intervention on Wound Pain and Wound Complications in Patients With Tonsillectomy: A Meta‐Analysis,” International Wound Journal 21, no. 4 (2024): e14619, 10.1111/iwj.14619.38152991 PMC10961898

The above article, published online on 28 December 2023, in Wiley Online Library (http://onlinelibrary.wiley.com/), has been retracted by agreement between the journal Editor in Chief, Professor Keith Harding; and John Wiley & Sons Ltd. Following an investigation by the publisher, all parties have concluded that this article was accepted solely on the basis of a compromised peer review process. The editors have therefore decided to retract the article. The authors did not respond to our notice regarding the retraction.

**RETRACTION:** J.‐L. Wu , Q. Zhang , L.‐H. Zhang , and J.‐Y. Li , “Effect of Comprehensive Nursing Intervention on Wound Pain and Wound Complications in Patients With Tonsillectomy: A Meta‐Analysis,” International Wound Journal 21, no. 4 (2024): e14619, 10.1111/iwj.14619.38152991 PMC10961898

The above article, published online on 28 December 2023, in Wiley Online Library (http://onlinelibrary.wiley.com/), has been retracted by agreement between the journal Editor in Chief, Professor Keith Harding; and John Wiley & Sons Ltd. Following an investigation by the publisher, all parties have concluded that this article was accepted solely on the basis of a compromised peer review process. The editors have therefore decided to retract the article. The authors did not respond to our notice regarding the retraction.

